# Host range and antibiotic resistance dissemination are shaped by distinct survival strategies of conjugative plasmids

**DOI:** 10.1093/nar/gkaf1479

**Published:** 2026-01-23

**Authors:** Ryuichi Ono, Naoki Konno, Yuki Nishimura, Chikara Furusawa

**Affiliations:** Innovative Genomics Institute, University of California, Berkeley, CA 94720, United States; Department of Comparative Biochemistry, University of California, Berkeley, CA 94720, United States; Department of Integrated Biosciences, Graduate School of Frontier Sciences, University of Tokyo, Kashiwa, Chiba 277-0882, Japan; Department of Biological Sciences, Graduate School of Science, University of Tokyo, Bunkyo-ku, Tokyo 113-0032, Japan; Universal Biology Institute, The University of Tokyo, 7-3-1 Hongo, Bunkyo-ku, Tokyo 113-0033, Japan; Department of Genetics, School of Medicine, Stanford University, 240 Pasteur Drive, Palo Alto, CA 94304, United States; Department of Integrated Biosciences, Graduate School of Frontier Sciences, University of Tokyo, Kashiwa, Chiba 277-0882, Japan; Universal Biology Institute, The University of Tokyo, 7-3-1 Hongo, Bunkyo-ku, Tokyo 113-0033, Japan; Center for Biosystems Dynamics Research, RIKEN, 6-7-1 Minatojima-minamimachi, Chuo-ku, Kobe 650-0047, Japan

## Abstract

Horizontal gene transfer is a major driver of bacterial evolution and the global dissemination of antibiotic resistance genes (ARGs). Conjugative plasmids play a crucial role in ARG spread across hosts within their host range, yet the genetic and functional determinants shaping plasmid host range remain poorly understood. Here, we systematically analyzed the gene content of conjugative/mobilizable plasmids derived from Enterobacterales from public databases and found that two distinct survival strategies were enriched in different host-range groups: a “stealth” strategy, which actively represses its own transcription by employing a global regulator *hns*, was particularly enriched in broad-host-range plasmids, whereas a “manipulative” strategy, which promotes its establishment by manipulating host machineries including SOS response and defense systems, was more common in narrow-host-range plasmids. Plasmids employing either strategy constituted the majority of conjugative plasmids analyzed, and accumulated significantly more ARGs than plasmids with neither strategy. Our data further suggested that stealth plasmids facilitate the acquisition of emerging ARGs, while manipulative plasmids amplify the copy number of established ARGs. This “stealth-first” model successfully recapitulated historical ARG dissemination patterns. These findings provide critical insights into the relationship between plasmid survival strategies and host range, advancing our understanding of the global patterns underlying plasmid-mediated ARG transmission.

## Graphical Abstract

**Graphical Abstract:**
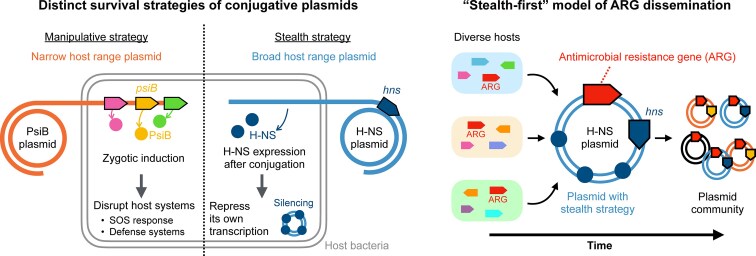

## Introduction

Horizontal gene transfer (HGT) plays a pivotal role in the remarkable adaptability and rapid evolution of prokaryotes [1, 2]. A significant proportion of HGT events are mediated by mobile genetic elements (MGEs), such as plasmids. HGT is a complex phenomenon driven by a trade-off between the selfish behavior of MGEs, which exploit host resources to increase their own copy number, and the potential benefits to the host from acquiring new genes [e.g. antibiotic resistance genes (ARGs)]. Previous studies have demonstrated that HGT is generally infrequent between evolutionarily distant host species or across disparate environments [3–5]. Additionally, the occurrence of HGT is often constrained by factors such as the presence of specific defense systems within hosts or abnormal protein–protein interactions (PPIs) between transferred and host proteins [6–8]. However, little is known about what genetic traits or adaptive mechanisms make certain MGEs particularly prone to facilitating HGT in natural environments. Understanding these mechanisms is not only essential for leveraging MGEs as tools for DNA delivery [9, 10] but also critically important for elucidating the emergence and spread of antibiotic resistance, an urgent global challenge.

According to estimates by the WHO, antibiotic-resistant bacteria were responsible for ~1.27 million deaths in 2019 [11]. Without appropriate measures, this number is predicted to swell to 10 million deaths annually by 2050 [12]. Furthermore, the World Bank has stated that the potential direct and indirect economic damage would be significant, with annual costs potentially rivaling those of the global financial crisis that began in 2008 [13]. The dissemination and acquisition of ARGs are heavily influenced by HGT mediated by MGEs [14]. Among these, conjugative plasmids—extrachromosomal DNA elements capable of transmitting between cells via conjugation—and mobilizable plasmids, which lack some components of the conjugation machinery but can be transferred using machinery provided by other MGEs, have been critical focal points of research. This is due to their significant role in the accumulation of ARGs [15, 16] and the ability to produce novel ARGs through mutagenesis triggered by conjugation [17, 18]. Another critical feature that underscores the importance of conjugative plasmids in ARG dissemination is their potential to broaden their host ranges. Some conjugative plasmids are capable of being transferred across phylogenetically distant species, even across phyla [19–21] or domains [22, 23]. Such broad host range often exceeds that of bacteriophages, which typically rely on highly specific (e.g. species- or strain-specific) interactions with host receptors for successful infection [24]. ARGs encoded on these broad-host-range (BHR) MGEs have the potential to spread not only across different bacterial species but also across diverse biological and ecological contexts, including human and animal hosts as well as environmental niches. As a result, they represent one of the most critical factors in the global dissemination of ARGs [25].

Against this backdrop, BHR plasmids remain a fascinating research focus to explore the dynamics of ARG dissemination. Recent advancements in computational methods have enabled more precise classification of plasmids and improved predictions of their host range [21]. Concurrently, various studies have revealed correlations between host range and genomic features, such as the number of restriction sites [26], and the number and structure of replication origins [27], suggesting that plasmids with different host ranges may require distinct strategies for adaptations. However, considering that plasmids serve as natural vehicles for gene transfer, a critical but largely unresolved question is how plasmid-encoded gene repertoires correspond to host range. Unraveling the distinct “survival strategies” that plasmids employ—encompassing not only transfer efficiency but also long-term persistence within a host—and understanding their implications for ARG dissemination remain largely unexplored.

In this study, we performed a comprehensive gene enrichment analysis between BHR and narrow-host-range (NHR) plasmids from Enterobacterales to systematically investigate how conjugative/mobilizable plasmids shape their host range and contribute to ARG dissemination in the enteric environment, where conjugation occurs frequently and clinically important lineages such as *Klebsiella pneumoniae* reside. We identified two genes, *hns* and *psiB*, that were strongly associated with BHR and NHR plasmids, respectively, and found to be almost mutually exclusive. Remarkably, plasmids carrying either gene represented the majority of conjugative/mobilizable plasmids in our dataset and exhibited a significantly greater tendency to accumulate ARGs than those lacking both. Further analyses focusing on conjugative plasmids revealed that H-NS plasmids—associated with a “stealth” strategy which dampens its own transcription—tend to acquire newly emerging ARGs, while PsiB plasmids—employing a “manipulative” strategy that manipulates host responses—preferentially disseminate ARGs already prevalent in microbial communities. A temporal analysis of 48 major ARGs uncovered a consistent, global trend in which novel ARGs are first acquired by stealth plasmids and subsequently spread via manipulative plasmids. This sequential dynamic supports a proposed “stealth-first” model of plasmid-mediated ARG dissemination. Together, these findings unveil distinct survival strategies that underpin plasmid host range and ARG dynamics, offering a new conceptual framework for understanding and predicting the evolutionary trajectories of antibiotic resistance.

## Materials and methods

### Datasets and gene annotation

Given the high conjugation frequency in the enteric environment [28] and the presence of clinically important lineages such as *Klebsiella pneumoniae*, Enterobacterales provides an ideal system for investigating the evolution of conjugation-based survival strategies. Therefore, a total of 943 complete plasmid genomes from Enterobacterales were used for the enrichment analysis. These genomes were derived from the 84th NCBI RefSeq database [29] and filtered by whether it had been assigned MOB types and host-range (Hrange) classifications in a prior study [21]. Notably, plasmids from Enterobacterales (943 sequences) accounted for the majority of the total plasmids (1874 sequences) with MOB types and host-range classifications in the dataset. Unlike the PLSDB and IMG/PR datasets used in subsequent analyses, which were restricted to conjugative plasmids, this dataset encompassed both conjugative and mobilizable plasmids. Additionally, on October 5, 2024, the PLSDB database (version: 2021_06_23_v2) [30, 31] was downloaded and used for subsequent analyses. For both datasets, GenBank files were retrieved via the NCBI Entrez API using the accession numbers indicated in the metadata files. For each CDS from the GenBank files, gene annotation was performed by running HMMscan from HMMER (version: 3.3) [32] against PFAM r35 with an *E*-value cutoff of 10⁻² (-E 1e-2). Plasmids were categorized as H-NS plasmid, PsiB plasmid, “Both” plasmid or “None” plasmid based on the presence or absence of genes annotated as “Histone_H-NS” (PF00816.24) and “PsiB” (PF06290.14). We note that our annotation pipeline assigns only the single best-hit Pfam domain (i.e. the one with the lowest *E*-value) to each protein. Consequently, this approach may overlook the functions of multi-domain proteins.

### Enrichment analysis

For the 943 plasmid genomes from Enterobacterales with MOB type and Hrange information, a previous study [21] assigned Hrange values based on the plasmid host range. “Single species” plasmids, which have the narrowest host range (Hrange = “I”), are restricted to a single bacterial species. Plasmids capable of transferring to multiple species but limited to those within the same family were assigned Hrange = “II”. Similarly, plasmids with Hrange values of “III”, “IV”, “V”, and “VI” were defined based on increasing host-range breadth. The broadest category, Hrange = “VI”, includes plasmids that can transfer to bacteria spanning multiple phyla. Plasmids with an Hrange of “I”, “II”, or “III” were classified as NHR plasmids (701 sequences), while those with an Hrange of “IV”, “V”, or “VI” were classified as BHR plasmids (242 sequences). To compare the frequency of each detected gene between NHR and BHR plasmids, Fisher’s exact test (fisher_exact from SciPy, version: 1.14.0) was conducted using HMMscan results. False Discovery Rate (FDR) for each hit was calculated by Benjamini–Hochberg procedure (false_discovery_control from SciPy, version: 1.14.0). To assess the statistical significance of the mutually exclusive distribution of *psiB* and *hns*, Chi-squared test (chi2_contingency from SciPy, version: 1.14.0) was conducted.

The goal of this analysis was to identify genes that characterize narrow or broad host ranges. However, enrichment analysis using all genes detected by HMMscan risks identifying genes specific to certain MOB types or even smaller subgroups, which may not reflect host-range characteristics. To address this, the analysis was restricted to genes present in at least 10% of plasmids within each host-range type and detected across a diverse set of MOB types. To measure the MOB-type distribution diversity of each gene, Simpson’s index was employed. For a more detailed explanation and an example, see Supplementary Discussion S1. The histograms of Simpson’s index for genes detected in BHR and NHR plasmids revealed three peaks near 0, 0.2, and 0.4 (Supplementary Fig. S1). The peak near 0 likely corresponds to genes specific to small plasmid subgroups within each host range, while the peaks near 0.2 and 0.4 represent genes more broadly distributed across plasmids within the same host range. To further investigate the distribution, we plotted the proportion of genes with Simpson’s index values exceeding a threshold *n* (0 ≤ *n* ≤ 1). A clear drop was observed near *n* = 0.4 in both BHR and NHR plasmids (Supplementary Fig. S1). Based on this observation, Simpson’s index threshold of 0.4 was applied to identify genes broadly distributed across plasmids for subsequent analyses. The results of the enrichment analysis for BHR and NHR plasmids, including each gene’s name, Simpson’s index, FDR, and MOB type composition, are provided in Supplementary Tables S1 and 2, respectively. Furthermore, representative genes that were excluded by Simpson’s index threshold are shown in Supplementary Table S3.

### Gene co-occurrence analysis

To assess how unusual the anti-co-occurrence of *hns* (335 plasmids) and *psiB* (295 plasmids) is among similarly prevalent genes, we calculated the Jaccard index for all gene pairs in which both genes were encoded by ≥250 plasmids out of the 943 plasmid genomes from Enterobacterales (Supplementary Fig. S2). To ensure this pattern was not an artifact of phylogenetic bias from oversampling, we performed two further validation steps. First, after down-sampling the dataset to one randomly selected plasmid per Plasmid Transfer Unit (PTU), the anti-co-occurrence between *hns* and *psiB* remained statistically significant (*P* = 0.035, Chi-squared test; Supplementary Fig. S3a). Second, the mutual exclusion was also found to be significant among plasmids encoding relaxases of either MOBP or MOBF, indicating this exclusivity cannot be solely explained by phylogenetic bias (Supplementary Fig. S3b). We also computed Jaccard indices for all gene pairs in the dataset and visualized the submatrices for the top 10 genes enriched in BHR and in NHR, respectively. Pairwise dissimilarities (1 − Jaccard) were hierarchically clustered (average linkage) using scipy.cluster.hierarchy (SciPy version: 1.14.0), and heatmaps/clustermaps were rendered with seaborn (version: 0.13.2) (Supplementary Fig. S4).

### Mixed-effects logistic regression of *hns*/*psiB* carriage across host-range levels

Using the host-range information assigned to the 943 plasmid genomes from Enterobacterales, we modeled the probability that a plasmid encodes *hns* or *psiB* as a function of host-range breadth using a binomial generalized linear mixed model (GLMM) with a logit link. Host-range categories I–VI were encoded as an ordinal numeric variable [1–6] and mean-centered prior to analysis so that the intercept corresponds to the average category. To account for potential bias introduced by oversampling of particular plasmid subgroups, we added a random intercept for MOB type as a proxy for subgroup structure, thereby absorbing subgroup-level baseline differences and within-group dependence. Plasmids labeled “Both” were treated as positive for each outcome when modeling *hns* and *psiB*, respectively. The model was specified in R’s formula notation as logit(p) ∼ Host range + (1 | MOB). This structure estimates the logistic regression coefficients for the fixed effect (Host range) on the presence/absence of *hns* or *psiB* (p), while the random effect (1 | MOB) fits a distinct intercept for each MOB type.

Models were fitted with glmer from lme4 (version: 1.1-37) with the bobyqa optimizer. The effect of host range was evaluated as the logistic regression coefficient of host range on the presence of *hns* or *psiB*. Two-sided *P*-values were obtained from Wald *z*-tests. Data import and wrangling used readr (version: 2.1.5) and dplyr (version: 1.1.4), and tidy summaries (including exponentiated coefficients) used broom.mixed (version: 1.1.4).

### Gene neighborhood analysis of DUF3085

We retrieved 82 GenBank records (.gb) from NCBI corresponding to contigs containing DUF3085 in our dataset consisting of the 943 plasmid genomes from Enterobacterales. For each record, coding sequences (CDS) were parsed in genomic order and annotated following the procedures described in Datasets and gene annotation. For each contig (treated as circular), we measured—for every CDS—the shortest gene-order distance to DUF3085 in CDS steps, allowing wrap-around (i.e. the smaller of the clockwise versus counterclockwise counts, strand ignored). We then pooled all contigs and, for each gene, calculated the mean distance to DUF3085 (Supplementary Fig. S5). For genes with a mean distance ≤ 10 CDS from DUF3085 (*n* = 9), we assessed potential complex formation with DUF3085 using the AlphaFold3 web server [33] (Supplementary Fig. S6). For each gene, the DUF3085 counterpart was the protein encoded on the same contig. Full-length amino-acid sequences were submitted as a two-chain (heteromeric) prediction with default server settings, and the top-ranked model per pair was retained for downstream inspection and reporting.

### Filtering and preliminary characterization of conjugative plasmids from PLSDB

All plasmids in PLSDB (version: 2021_06_23_v2) were analyzed using MOB-typer (version: 3.1.9) [34]. Plasmids classified as “conjugative” with sequence lengths of 500 kb or less were used for downstream analyses (hereafter referred to as PLSDB plasmids). Sequence length and GC content were obtained from metadata provided by PLSDB. IS content (%) was calculated for all PLSDB plasmids using ISEScan (version: 1.7.2.3) [35]. Chi-squared test (chi2_contingency from SciPy, version: 1.14.0) was conducted to assess the statistical significance of the mutually exclusive distribution of PsiB and H-NS. To compare the distribution of GC content, length, IS content, ARG number per 100 kb, and integron content, Mann–Whitney *U* test (mannwhitneyu from SciPy, version: 1.14.0) was conducted. FDR was calculated by Benjamini–Hochberg procedure (false_discovery_control from SciPy, version: 1.14.0).

### Plasmid network based on proteome similarity

To construct a plasmid proteome similarity network, we applied a previously proposed method [21, 36]. From the PLSDB dataset comprising 10 827 conjugative plasmids, 1000 plasmid sequences were randomly sampled using the sample command of SeqKit [37] (version: 2.9.0). The network was generated with AccNet (accnet.pl, version 1.2) using the parameters –threshold 1.1 –kp “-s 1.12 -e 1e-14 -c 0.8”. In this framework, proteins encoded on plasmids were clustered into homologous protein clusters (HPCs) if they shared >30% amino acid identity and >80% alignment coverage. The bipartite network of plasmids and HPCs output by AccNet was visualized in Gephi [38] (version: 0.10.1) using the ForceAtlas2 [39] layout algorithm. The scaling parameter was set to 1, edge weight was set to null, and the algorithm was run for 6930 iterations followed by an additional 129 iterations with the “Prevent Overlap” option enabled.

### Annotating F*rpo* promoters

F*rpo* promoters were detected in the PLSDB dataset by conducting BLAST searches using a set of five well-characterized Frpo promoter sequences together with 13 putative Frpo promoter sequences identified in a large-scale analysis (obtained from Supplementary Table S2 of [40]). Searches were performed with BLASTn (version: 2.16.0) using an *E*-value threshold of 1 × 10^−6^, while all other parameters were kept at their default settings. To compare the distribution of plasmids with F*rpo* promoters, Chi-squared test (chi2_contingency from SciPy, version: 1.14.0) was conducted. FDR was calculated by Benjamini–Hochberg procedure (false_discovery_control from SciPy, version: 1.14.0). Due to floating-point underflow in SciPy, *P*-values smaller than machine precision are reported as <10^−300^.

### ARG and integron annotation

AMRFinderPlus (version: 3.12.8, AMRFinder database version: 2024-07-22.1) [41] was used for PLSDB plasmids to detect ARGs. Only ARGs with the annotation of “Element type = AMR” were included in downstream analyses. Integron regions within each plasmid were annotated using IntegronFinder 2.0 [42]. Detected ARGs were classified into three categories based on their prevalence in the dataset. ARGs encoded by fewer than 10 plasmids were classified as “Rare,” those found in 10 to 99 plasmids as “Moderate,” and those present in 100 or more plasmids as “Prevalent.” We validated this categorization by computing Simpson’s diversity index over MOB type for each ARG (as in the Enrichment analysis) and observing a monotonic increase from “Rare” to “Prevalent” categories (Supplementary Fig. S7), indicating broader plasmid-type representation for more prevalent ARGs. For each ARG, if its start and end positions were entirely contained within an integron region, it was annotated as an ARG encoded within an integron. To compare the distribution of Simpson’s index of ARGs across prevalence categories, Mann–Whitney *U*-test (mannwhitneyu from SciPy, version: 1.14.0) was conducted. FDR was calculated by Benjamini–Hochberg procedure (false_discovery_control from SciPy, version: 1.14.0). To compare the distribution of the proportions of plasmid types carrying each ARG, Wilcoxon rank-sum test (wilcoxon from SciPy, version: 1.14.0) was conducted. FDR was calculated by Benjamini–Hochberg procedure (false_discovery_control from SciPy, version: 1.14.0). To compare the distribution of “Rare” and “Moderate” ARGs across different plasmid types, Chi-squared test (chi2_contingency from SciPy, version: 1.14.0) was conducted. FDR was calculated by Benjamini–Hochberg procedure (false_discovery_control from SciPy, version: 1.14.0).

### The time-course analysis of ARG dissemination

The “CollectionDate_BIOSAMPLE” column from PLSDB metadata was manually inspected to determine the year each plasmid was reported. When multiple years were listed (e.g. 1950/1955), the older year was used by default. However, when the older year was before 1900, the more recent year was used (e.g. 1800/2014 or 1900/1967). Plasmids lacking values in the “CollectionDate_BIOSAMPLE” column or with unclear date annotations were excluded from time-course analyses. Threshold Year was defined as the year at which the number of detections for each plasmid type reached the first quartile of their respective detection counts as of 2021. It was calculated for each plasmid type, excluding Both, using all ARGs that were encoded by >100 plasmids (*n* = 48). To compare the distribution of Threshold Year values, the Wilcoxon rank-sum test (Wilcoxon from SciPy, version: 1.14.0) was conducted. The FDR was calculated using the Benjamini–Hochberg procedure (false_discovery_control from SciPy, version: 1.14.0).

### Filtering and preliminary characterization conjugative plasmids reconstructed from metagenomes (IMG/PR)

We filtered the IMG/PR database (release 2023-08-08_1) and retained only plasmid records recovered from metagenomes, excluding entries labeled “Isolate” or “Single amplified genome (SAG).” We further required the IMG/PR quality flag “putatively complete = Yes,” “length ≤ 500 000,” “putative_phage_plasmid = No,” and evidence of conjugative machinery, operationalized as the presence of at least one relaxase (MOB) annotation and one type IV coupling protein (T4CP) annotation on the same contig. Gene predictions were taken as provided by IMG/PR. Applying these criteria yielded 11 160 metagenome-derived, putative conjugative plasmids for downstream analyses. To identify plasmids encoding *hns* or *psiB*, we used profile HMMs corresponding to PF00816.26 (*hns*) and PF06290.16 (*psiB*) downloaded from InterPro on 21 June 2025. All predicted proteins from the filtered IMG/PR plasmids were scanned with hmmsearch from HMMER (version: 3.3) with an *E*-value cutoff of 10⁻² (-E 1e-2). A plasmid was typed as “H-NS” or “PsiB” if at least one protein on the contig matched the corresponding profile at or below this threshold; plasmids matching both profiles were labeled “Both,” and those matching neither were labeled “None.” Chi-squared test (chi2_contingency from SciPy, version: 1.14.0) was conducted to assess the statistical significance of the mutually exclusive distribution of *psiB* and *hns*. To compare the distribution of GC content and length, Mann–Whitney *U* test (mannwhitneyu from SciPy, version: 1.14.0) was conducted. FDR was calculated by Benjamini–Hochberg procedure (false_discovery_control from SciPy, version: 1.14.0).

## Results

### *hns* and *psiB* characterize plasmids with broad and narrow host ranges, respectively

**Figure 1.**
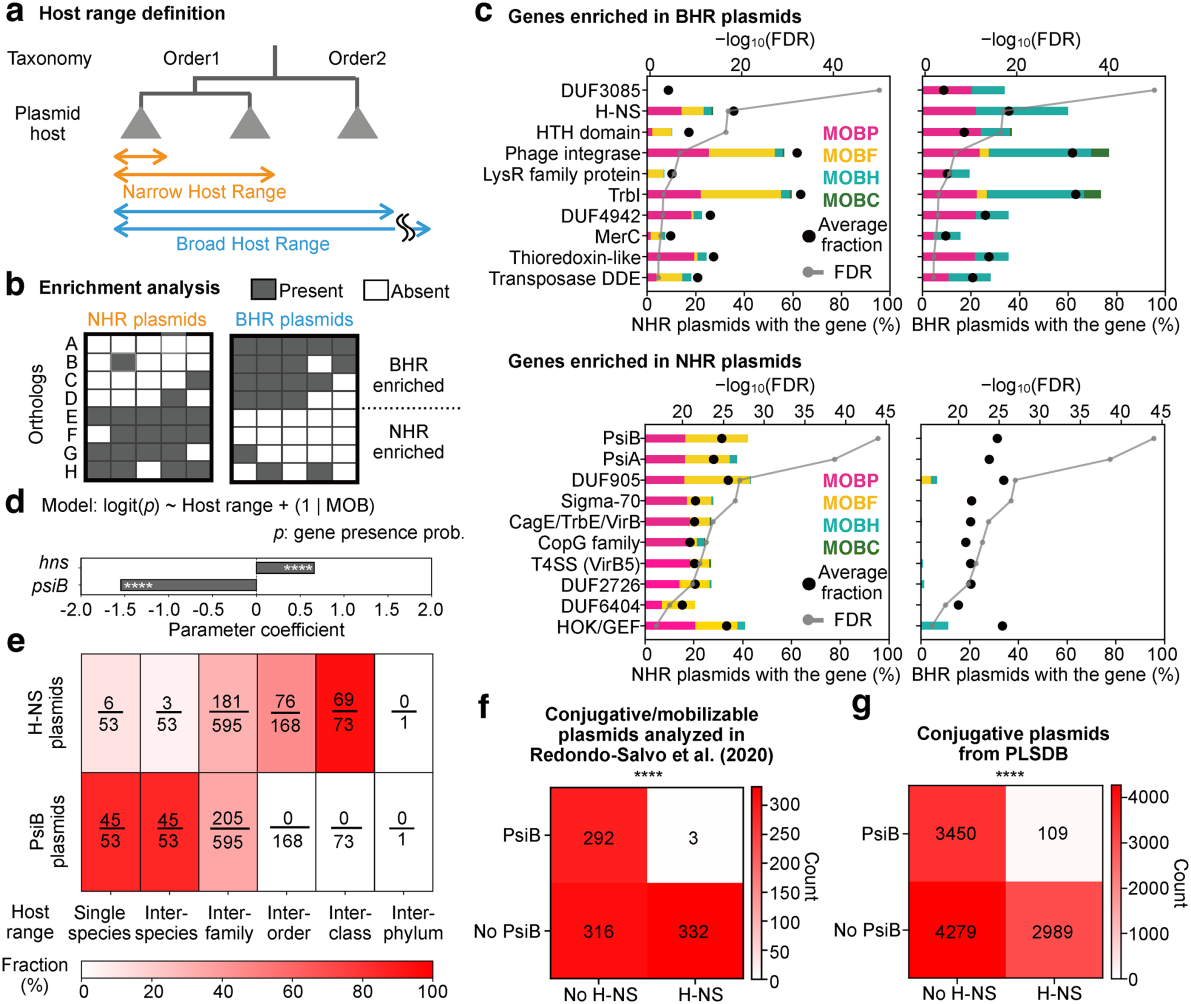
*hns* and *psiB* exhibit mutually exclusive distributions and are characteristic of BHR and NHR plasmids, respectively. (**a**) Schematic diagram of the host-range definition used in this study. (**b**) Schematic diagram of the enrichment analysis. Orthologs (A–D) are enriched in BHR plasmids, whereas (E–H) are enriched in NHR plasmids. Gray squares indicate presence of orthologs in each plasmid; white squares indicate absence. (**c**) Results of the enrichment analysis. Genes were ranked in ascending order of *P*-values from the enrichment analysis among those present in at least 10% of plasmids within each host range and distributed across multiple MOB types (see Materials and methods). Bar colors correspond to the MOB types of the plasmids encoding each gene, while bar heights represent the proportion of plasmids within each host range that carry the gene. Black circles indicate the proportion of plasmids that contain the gene among all analyzed plasmids. For each hit, FDR was calculated and -log_10_(FDR) of was shown in gray line. (**d**) The logistic regression coefficients of the host range on the presence of *hns* or *psiB* are shown as bar plots (see Materials and methods). Significance is indicated with asterisks (****P < 0.0001, Wald *z*-test). (**e**) Proportion of plasmids encoding *hns* or *psiB* across different host-range categories. “Single species” plasmids have the narrowest host range, being restricted to a single bacterial species, whereas “Inter-phylum” plasmids have the broadest host range, spanning multiple bacterial phyla. (**f** and **g**) Contingency tables showing the distribution of *hns* and *psiB* across plasmids. (f) 943 conjugative/mobilizable plasmids from Enterobacterales annotated by Redondo-Salvo *et al.* (2020). (g) 10 827 conjugative plasmids from PLSDB. Rows indicate the presence or absence of *psiB*, and columns indicate the presence or absence of *hns*. The numbers within the boxes indicate the count of plasmids in each category, with color intensity corresponding to plasmid counts. Significance is shown with asterisks (****P < 0.0001, Chi-squared test).

To elucidate the mechanisms by which plasmids acquire either BHR or NHR characteristics, we conducted a gene enrichment analysis between BHR and NHR conjugative and mobilizable plasmids (Fig. 1 a and b). BHR plasmids are defined as those capable of transferring across multiple bacterial taxa (Orders), whereas NHR plasmids are limited to transfer within a single order or a more restricted taxonomic group. We focused our analysis on 943 conjugative and mobilizable plasmids derived from Enterobacterales (701 NHR and 242 BHR plasmids).

Distinct patterns of gene enrichment were observed between BHR and NHR plasmids (Fig. 1c). Expectedly, BHR plasmids were enriched with genes associated with HGT and conjugation. For example, they were enriched with genes central to the integron system, such as integrase (PF00589.25), which facilitates the capture and expression of foreign genes [43, 44]. Other examples include *trbI* (PF03743.17), a protein that increases mating efficiency by up to 20-fold during conjugative transfer [45, 46]. We further observed that *hns* (PF00816.24), a global transcriptional regulator that modulates gene expression and chromosomal structure [47], had the second-lowest *P*-value in our enrichment analysis, following only the uncharacterized gene DUF3085 (PF11284.11). *hns* was both enriched and highly prevalent in BHR plasmids, being encoded in ~60% of them, whereas it is found in <30% of NHR plasmids. Among genes enriched in BHR plasmids, these three genes tended to co-occur on the same plasmids (Supplementary Fig. S4a). Considering their prevalence and enrichment among BHR plasmids, these genes, especially *hns*, can be considered as a suitable marker for BHR plasmids.

On the other hand, NHR plasmids were associated with genes such as *psiB* (PF06290.14) and *psiA* (PF06952.14). Both *psiB* and *psiA* are known to inhibit the SOS response, particularly during plasmid conjugative transfer. Especially, *psiB* is a well-studied gene, which is known to function independently [48]. Additionally, DUF905 (PF06006.15) is often encoded near these genes, suggesting potential functional relationships with them [17, 18, 40, 49–51]. Many of the genes enriched in NHR plasmids tended to co-occur on the same plasmids (Supplementary Fig. S4b). Considering the co-occurence with other genes and clear enrichment, *psiB* has been chosen as a marker gene for NHR plasmids.

Previous literature that found *hns* and *psiB* from some BHR and NHR plasmids, respectively [17, 52], reinforce the idea of using them as markers of host range. In addition to that, We found that the proportion of plasmids encoding *hns* increases as the host range broadens, while psiB exhibits the opposite trend (regression coefficient = 0.6593, *P* = 4.34 × 10^−7^, regression coefficient = −1.5449, *P* = 2.19 × 10⁻¹³, respectively; Wald *z*-test) (Fig. 1d and e). Moreover, these genes were distributed almost mutually exclusively (Fig. 1f). Jaccard index between the distribution of *hns* and *psiB* was 4.78 × 10⁻^3^, which was the 17th smallest out of 990 gene pairs analyzed (Supplementary Fig. S2). Such an exclusive distribution was robustly observed in a comprehensive analysis of all conjugative plasmids (10 827 sequences) available in the PLSDB database [30, 31] (Fig. 1g), and a similar trend was also present in 11 160 putative conjugative plasmids reconstructed from metagenomes (Supplementary Fig. S8).

### H-NS and PsiB plasmids distributed across diverse plasmid taxa and have undergone distinct adaptations

The mutually exclusive distribution of these genes could be an artefact of phylogenetically (taxonomically) biased distribution across diverse plasmid taxonomy. To rule out this possibility, we next examined the taxonomic group of plasmids carrying these genes. Inferring accurate phylogenetics of plasmid lineages is challenging due to frequent recombination events with host genomes and other MGEs, as well as the absence of essential genes. However, MOB typing, which classifies plasmids based on the sequence of relaxase—a protein essential for conjugative transfer and present in all mobilizable and conjugative plasmids—provides a classification scheme of plasmid lineages [53–55]. The sequences of relaxase with different MOB types differ substantially and rarely coexist on a single plasmid. Moreover, relaxase evolves very slowly compared to other features such as plasmid size or other encoded genes [54]. These facts make it possible to assume that plasmids belonging to different MOB types are phylogenetically more distant from each other than those within the same MOB type.

**Figure 2.**
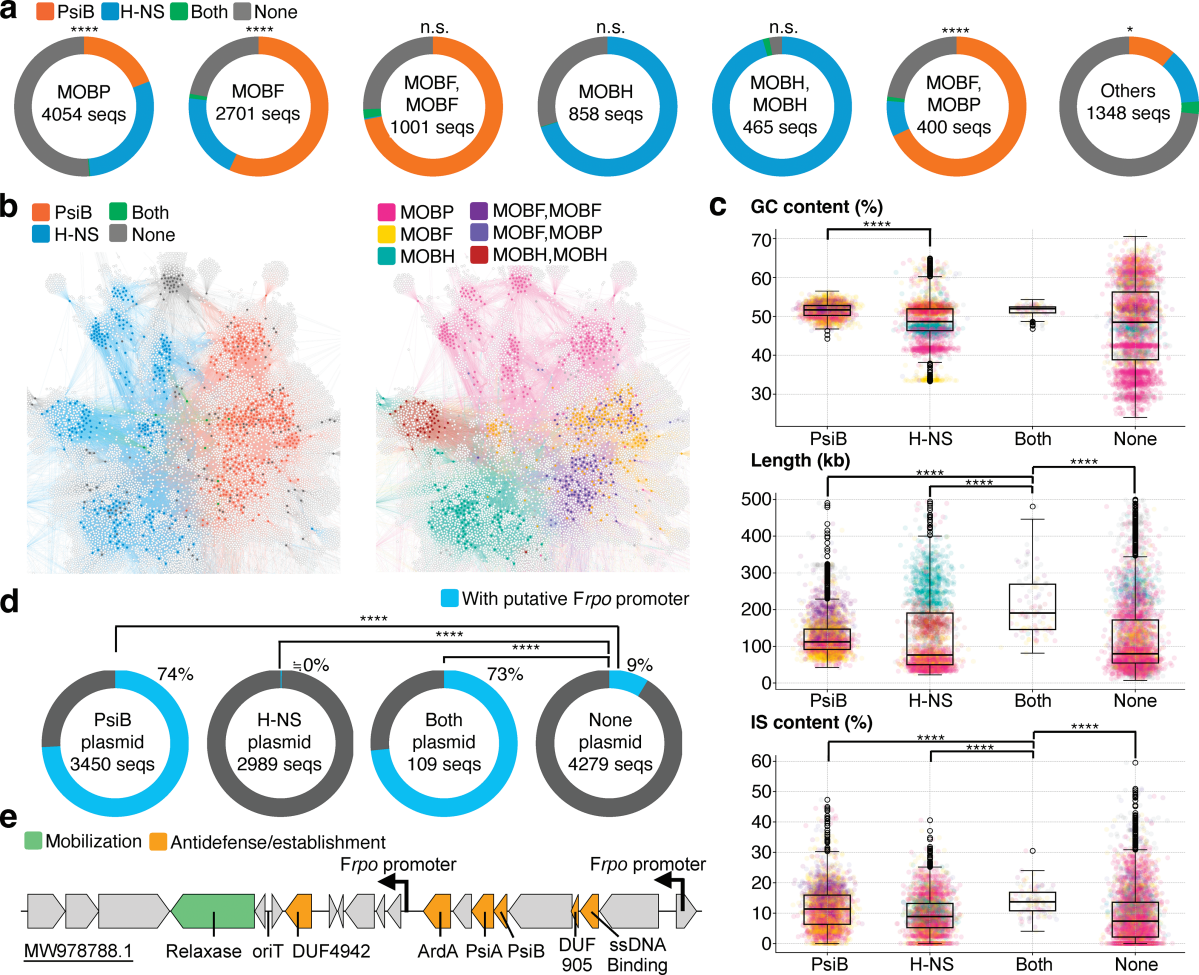
H-NS and PsiB plasmids originate from diverse plasmid clades and exhibit distinct adaptations. (**a**) Proportion of H-NS and PsiB plasmids for each MOB type among all analyzed conjugative plasmids (10 827 sequences). The six most abundant MOB types, as well as the sum of all remaining MOB types, are shown. Significance is shown with asterisks (*P < 0.05; ****P < 0.0001, Chi-squared test). (**b**) Bipartite network linking plasmids (colored nodes) and HPCs (white nodes) encoded on them. An edge indicates that a plasmid encodes ≥1 protein assigned to the HPC. Left, plasmids colored by plasmid type. Right, plasmids colored by MOB type. Layout was computed with ForceAtlas2 (see Materials and methods). The full network is shown in Supplementary Fig. S9. (**c**) Characteristics of each plasmid type. Dot colors correspond to plasmid MOB types, with color assignments matching those in Fig. 2b. Significance is shown with asterisks (****FDR < 0.0001, Mann–Whitney *U*-test). (**d**) Proportion of plasmids carrying an F*rpo* promoter, by plasmid type. Significance is shown with asterisks (****FDR < 0.0001, Chi-squared test). (**e**) Example of a putative leading region from a PsiB plasmid. Relaxase and oriT (origin of Transfer) were annotated using MOB-suite, while other genes were annotated using HMMER (see Materials and methods).

Using 10 827 conjugative plasmids from PLSDB, we analyzed the MOB-type compositions of plasmids in our dataset (Fig. 2a). H-NS and PsiB plasmids were distributed across diverse MOB types, which indicates that *hns* and *psiB* have been independently acquired by various clades. Notably, certain MOB types, such as MOBP and MOBF, contained both H-NS plasmids and PsiB plasmids, and their mutually exclusive distribution was statistically significant (Fig. 2a). The fact that a single taxonomic group of plasmids contains both types of plasmids, yet the number of plasmids with both genes remains very small, implies that this exclusivity cannot be solely explained by phylogenetic bias. Instead, it suggests a nontrivial evolutionary pattern driven by distinct selective pressures.

To investigate whether this plasmid type classification reflects a deeper organization of the entire gene repertoire that spans across diverse backbones, we constructed a plasmid network based on proteome similarity. On this network, H-NS plasmids and PsiB plasmids each formed distinct clusters (Fig. 2b). Coloring the same network by MOB type showed that plasmids within the same MOB group tended to cluster, indicating that backbone features partly shape proteome composition. Nevertheless, the H-NS and PsiB clusters spanned multiple MOB types, indicating that the H-NS/PsiB classification captures an axis of proteomic organization that is independent of backbone type.

We next examined the general sequence characteristics of plasmids carrying *hns* or *psiB*, as well as those carrying neither or both genes (“None” and “Both”). H-NS plasmids exhibited notably lower GC contents compared to PsiB plasmids (Mann–Whitney *U*-test, FDR = 4.44 × 10^−196^) (Fig. 2c). Given that *hns* is a transcriptional repressor with a preference for AT-rich DNA [56, 57], this suggests sequence-level adaptation for transcriptional regulation centered around *hns*. This possibility is reinforced by the observation that only H-NS plasmids harbor distinct subgroups of extremely AT-rich MOBP and MOBF plasmids, whereas PsiB plasmids within the same MOB families typically retain moderate GC levels. In other words, plasmids with otherwise similar backbones can follow divergent evolutionary paths depending on whether they encode *hns* or *psiB*. Furthermore, each plasmid type exhibited distinct size distributions. “Both” plasmids (median = 190 kbp) had a significantly larger size distribution, with a median 1.69 times greater than that of the next largest group, PsiB plasmids (median = 112 kbp) (Fig. 2c). The scarcity of “Both” plasmids, along with the fact that they are larger and contain more genes per plasmid compared to other groups, suggests that these plasmids may have acquired sequences from multiple plasmids through recombination or adopted a unique mechanism that promotes the accumulation of diverse genes. Interestingly, these size differences mirrored the proportion of insertion sequences within their nucleotide sequences, suggesting that IS elements act as additional drivers of plasmid size variation (Fig. 2c).

An additional characteristic feature of conjugative plasmids is the leading region, defined as the segment that enters the recipient cell first as single-stranded DNA during conjugation. Previous studies have shown the importance of this region for the outcome of conjugative transfer. In particular, some plasmids encode specialized promoters (F*rpo* promoters) within their leading region that allow transcription from single-stranded DNA [58, 59], resulting in the early expression of genes in this region even before conjugation is complete. Leading regions are frequently enriched in genes such as anti-defense factors that protect plasmids from host defense machineries, thereby increasing both the efficiency of conjugative transfer and the likelihood of subsequent plasmid maintenance [40, 49].

We next asked whether this unique and effective machinery—leading region-based establishment—is a feature shared by both H-NS and PsiB plasmids, or whether it is specific to one of these groups. To address this, we searched the PLSDB dataset for the F*rpo* promoter, which is a characteristic of this system. Interestingly, as many as 74% of PsiB plasmids contained one or more F*rpo* promoters, whereas F*rpo* promoters were observed in only seven H-NS plasmids. Compared to “None” plasmids, PsiB plasmids showed a significant enrichment of leading regions (Chi-squared test, FDR < 10^−300^). Conversely, H-NS plasmids exhibited a striking depletion of leading regions (Chi-squared test, FDR = 6.66 × 10^−57^) (Fig. 2d). An example of a putative leading region is shown in Fig. 2e. In this case, the region located downstream of the oriT corresponds to the putative leading region, in which CDSs are arranged in the direction transcribed from the F*rpo* promoters. Notably, *psiB* itself is included within the leading region, consistent both with our observation that this system is characteristic of PsiB plasmids and with a previous report that identified an enrichment of *psiB* in leading regions [40].

### Both H-NS plasmids and PsiB plasmids are significant contributors to the dissemination of ARGs

**Figure 3.**
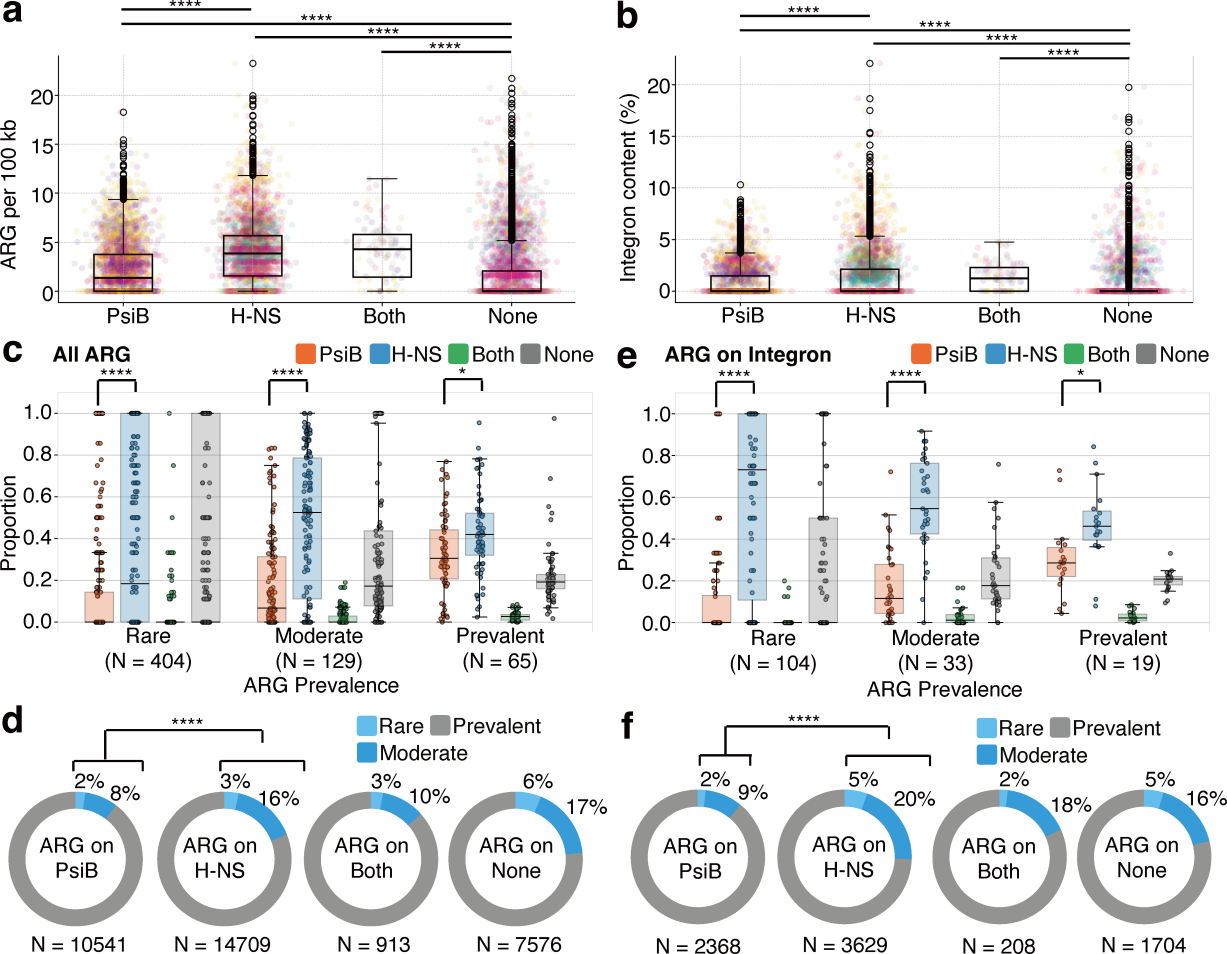
Conjugative plasmids carrying *hns* or *psiB* are major contributors to ARG dissemination. (**a** and **b**) Number of ARGs per 100 kb in each plasmid type. Dot colors correspond to plasmid MOB types, with color assignments matching those in Fig. 2b. Significance is shown with asterisks (****FDR < 0.0001, Mann–Whitney *U*-test). (**c** and **e**) Proportion of plasmids carrying ARGs by plasmid type and ARG prevalence. ARGs were categorized based on the number of plasmids encoding them: ARGs present in fewer than 10 plasmids were classified as “Rare,” those found in 10 to 99 plasmids as “Moderate,” and those present in 100 or more plasmids as “Prevalent.” The numbers of unique ARGs within each prevalence category are shown in the plot. Significance is shown with asterisks (*FDR < 0.05; ****FDR < 0.0001, Wilcoxon rank-sum test). (**d** and **f**) Proportions of ARGs in each prevalence category detected in each plasmid type. The total numbers of ARGs detected in each plasmid type are shown in the plot. Significance is shown with asterisks (****FDR < 0.0001, Chi-squared test). (a, c, and d) Analysis on all detected ARGs. (b, e, and f) Analysis on ARGs located within integron regions.

These aforementioned analyses indicate that H-NS and PsiB plasmids represent two evolutionarily differentiated types with distinct genetic features. To assess how each type influences the spread of ARGs, we analyzed ARG accumulation across the two types (Fig. 3a). Interestingly, PsiB plasmids, H-NS plasmids, and “Both” plasmids accumulated more ARGs than “None” plasmids (Mann–Whitney *U*-test, FDR = 4.13 × 10^−74^, 2.88 × 10^−288^, and 7.22 × 10^−26^, respectively). H-NS plasmids accumulated significantly more ARGs than PsiB plasmids (Mann–Whitney *U*-test, FDR = 2.67 × 10⁻^109^), the median number of ARGs normalized by plasmid size being 2.87 times higher in H-NS plasmids that in PsiB plasmids. These results suggest that, in the context of ARG dissemination by conjugative plasmids, plasmids carrying *hns* and/or *psiB* play a significantly more important role compared to those lacking these genes. Since integrons are known to play a critical role in the acquisition of ARGs in MGEs [60], we examined the proportion of integron regions in the sequences of each plasmid belonging to each plasmid type (Fig. 3b). As expected, PsiB plasmids, H-NS plasmids, and “Both” plasmids contained a higher proportion of plasmids with integron regions compared to “None” plasmids (Mann–Whitney *U*-test, FDR = 3.14 × 10^−61^, 1.09 × 10^−144^, and 8.47 × 10^−43^, respectively). Additionally, H-NS plasmids contained more plasmids with integron regions than PsiB plasmids (Mann–Whitney *U*-test, FDR = 6.94 × 10^−24^). This pattern aligns with the trends observed for the number of ARGs per 100 kbp.

We then examined the characteristics of ARGs found on each plasmid type. The detected ARGs in the dataset were classified into three categories based on the number of plasmids encoding each ARG. ARGs encoded by fewer than 10 plasmids were classified as “Rare,” those found in 10 to 99 plasmids as “Moderate,” and those present in 100 or more plasmids as “Prevalent.” For each prevalence category, we plotted the proportion of plasmid types encoding each ARG. The results indicate that although H-NS plasmids generally carry ARGs more frequently than PsiB plasmids, the difference in ARG carriage frequency between the two plasmid types becomes smaller for prevalent ARGs (Fig. 3c). Furthermore, among the ARGs detected in H-NS plasmids, the proportion classified as “Rare” or “Moderate” was higher than in PsiB plasmids (Chi-squared test, FDR = 8.31 × 10⁻⁸¹) (Fig. 3d). These data suggest that rare ARGs, which have not yet widely spread within a population, are preferentially acquired by H-NS plasmids. The trend became even more pronounced when only the ARGs located within integron regions (Fig. 3e and f) were analyzed. These results suggest that H-NS plasmids tend to capture ARGs that have not yet been widely spread in the population, possibly facilitated by integrons.

### H-NS plasmids acquire ARGs earlier than other conjugative plasmids

**Figure 4.**
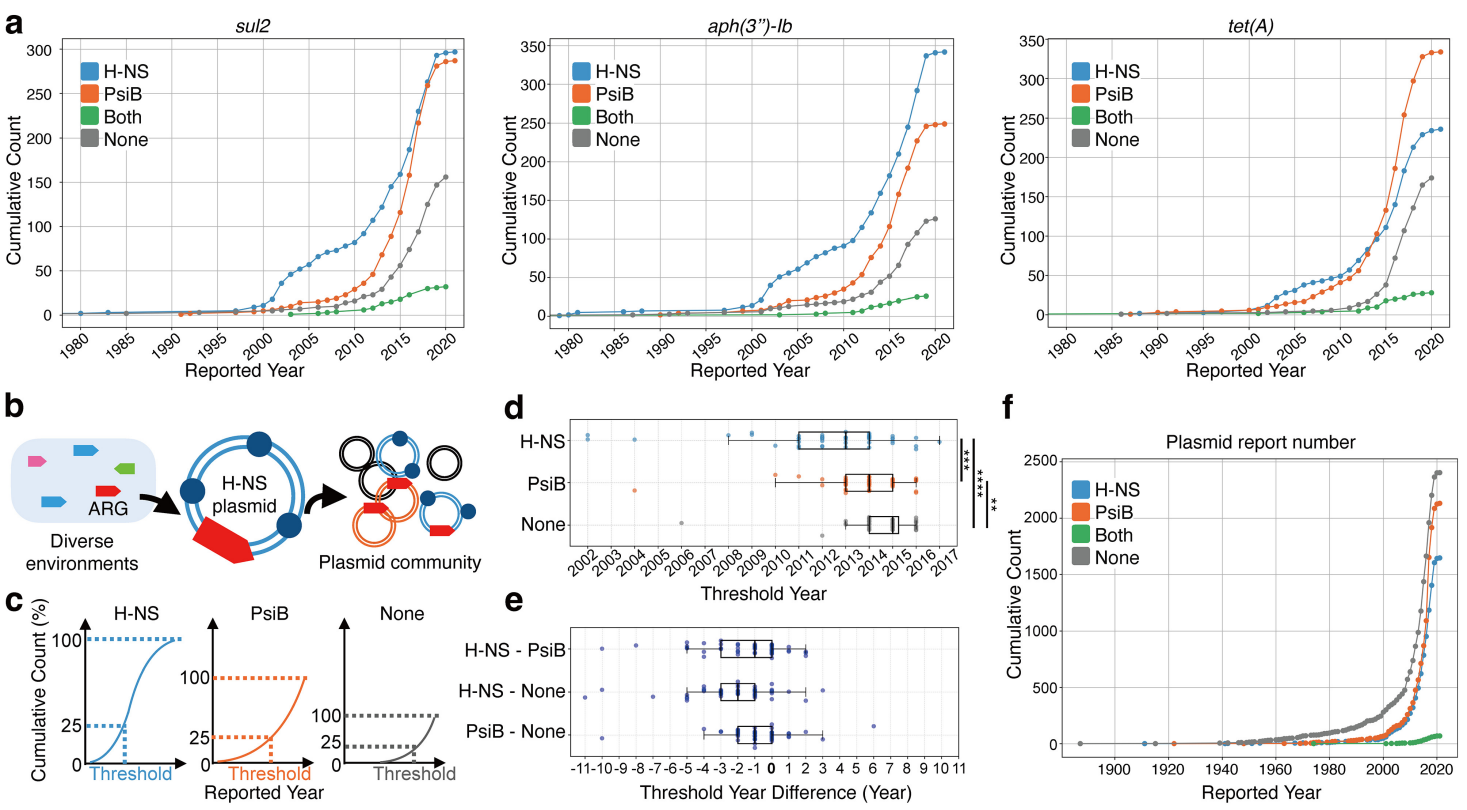
ARG acquisition by H-NS plasmids precedes that by PsiB plasmids. (**a**) Cumulative frequency curves illustrating the temporal increase in the number of plasmid records encoding *sul2, aph(3′')-Ib*, and *tet (A)* based on the collection dates registered in the PLSDB database (see Materials and methods). (**b**) Schematic diagram of the model of ARG spread mediated by H-NS plasmids. (**c**) Schematic diagram illustrating the calculation method of the Threshold Year. (**d**) Distribution of Threshold Year values for all ARGs that were encoded by >100 plasmids (*n* = 48). Significance is shown with asterisks (**FDR < 0.01; ***FDR < 0.001; ****FDR < 0.0001, Wilcoxon rank-sum test). (**e**) Distribution of the differences in Threshold Year values between plasmid types for the same ARG, calculated for all ARGs encoded by >100 plasmids (*n* = 48). (**f**) Cumulative frequency curves showing the numbers of reported plasmid records by collection year for each plasmid type.

Our findings so far suggest that H-NS plasmids contribute to the acquisition of rare or emerging ARGs. If this hypothesis is correct, the acquisition of ARGs by H-NS plasmids is expected to precede their acquisition by PsiB plasmids. To test this, we analyzed all ARGs that were encoded by >100 plasmids (*n* = 48), focusing in particular on the temporal distribution of plasmids carrying the same ARG. For illustrative purposes, we first highlight the top 10 most frequently detected ARGs in the dataset. For all of these top 10 ARGs except *mph(A)* and *aadA1*, a sharp increase in the detection of ARGs on H-NS plasmids preceded the corresponding increase on PsiB plasmids (Fig. 4a and Supplementary Fig. S10). These findings support a model in which H-NS plasmids, which tend to exhibit a broad host range, act as hubs for the early acquisition and dissemination of ARGs by conjugative plasmids. They likely achieve this by capturing ARGs from diverse environments and subsequently distributing them within the plasmid community (Fig. 4b). To assess whether this trend holds across a broader range of ARGs, we compared the distribution of “threshold years”—the year at which each plasmid type reached the first quartile of its cumulative detection count as of 2021—for all 48 ARGs (Fig. 4c). The resulting distribution showed that the acquisition of ARGs by H-NS and PsiB plasmids significantly preceded that by “None” plasmids (Wilcoxon rank-sum test, FDR = 1.42 × 10^-6^ and 1.89 × 10⁻³, respectively), and that acquisition by H-NS plasmids preceded that by PsiB plasmids (FDR = 1.70 × 10^-4^) (Fig. 4d). This trend—namely, the precedence of H-NS and PsiB plasmids over “None” plasmids, as well as the precedence of H-NS plasmids over PsiB plasmids—was consistently observed when calculating the differences in Threshold Years across plasmid types for each ARG (Fig. 4e). It indicates that, both for individual ARGs and as an overall trend, the timing of ARG acquisition on H-NS plasmids precedes that on PsiB plasmids. Moreover, throughout the entire period, the number of PsiB plasmids reported in PLSDB was consistently similar to that of H-NS plasmids, with PsiB plasmids being slightly more prevalent, confirming that the observed tendency for ARG acquisition by H-NS plasmids to precede that of PsiB plasmids is not attributable to sampling bias (Fig. 4f).

## Discussion

In this study, we suggested that *hns* and *psiB* can be used as markers for BHR and NHR plasmids, respectively, and found that the proportion of plasmids carrying each gene changes significantly with host-range breadth (Fig. 1c–e). Importantly, H-NS plasmids and PsiB plasmids behave as two contrasting—and largely mutually exclusive—types: despite occurring on diverse plasmid backbones, the two genes rarely co-occur (Figs 1f, g, and 2a), and this classification captures a convergence at the proteome level that spans diverse backbones: across diverse backbones, proteomes cluster into two types corresponding to H-NS and PsiB plasmids (Fig. 2b). In parallel, relative to “None” plasmids, PsiB plasmids are strikingly enriched for the F*rpo* promoter and achieve leading-region–based establishment via zygotic induction during conjugation, whereas H-NS plasmids carry F*rpo* significantly less often than “None” plasmids and instead show signatures of self-repression: H-NS plasmids tend to be more AT-rich, which facilitates binding by H-NS—a global repressor that prefers AT-rich DNA—and thereby promotes self-repression of plasmid-encoded transcription (Fig. 2c and d). Together, these sharp contrasts indicate that the two plasmid classes have evolved fundamentally different modes of adaptation, corresponding to distinct “survival strategies.” We adopt this concept to emphasize a life-cycle perspective that extends beyond the transfer event itself, encompassing not only mechanisms for successful conjugation but also subsequent adaptations for long-term persistence. These distinct strategies appear finely tuned to the characteristic host-range regimes of each plasmid class.

Mechanistically, PsiB plasmids can be characterized by F*rpo*-driven zygotic induction in the recipient cell (Fig. 2d). *psiB* itself suppresses the SOS response during conjugative transfer, and prior work shows that genes transcribed from the leading region are enriched for anti-defense functions [40, 49]. Together, these observations are consistent with a “manipulative strategy” in which early-expressed loci dampen host stress responses and defense pathways to promote plasmid establishment. This view aligns with the tendency of PsiB plasmids to be NHR, as effective manipulation often requires specific PPIs with host factors and thus limits portability across taxa; accordingly, *psiB* activity has been reported to operate over a restricted set of susceptible bacterial hosts [17, 50].

The mechanisms underlying the H-NS plasmid strategy are particularly intriguing, as the pleiotropic nature of its key marker, *hns*. Beyond regulating chromosomal transcription of its host, plasmid-encoded *hns* has several functions that underpin the “stealth” strategy. These pleiotropic effects include its role as a xenogeneic silencer that represses foreign AT-rich DNA, its ability to modulate plasmid-specific processes such as conjugation, and crucially, its proposed ability to minimize the fitness cost and toxicity associated with plasmid acquisition (see Supplementary Discussion S2 for a detailed review and references). These observations, together with our finding that H-NS plasmids are more AT-rich and carry F*rpo* less often, support a model in which plasmid-encoded *hns* binds AT-rich segments on the plasmid to dampen its own transcription and thereby lower the fitness cost in recipients upon plasmid acquisition. However, it should be noted that while *hns* carriage provides an effective means to reduce fitness costs, this does not definitively establish that H-NS plasmids impose a lower cost compared to PsiB plasmids. For instance, the lag time before recipient growth resumes post-conjugation is highly relevant to the fitness cost of plasmid acquisition [61]. *psiB*-mediated inhibition of the SOS response could plausibly contribute to shortening this lag time, thereby potentially reducing the overall acquisition cost. Thus, while we cannot provide a conclusive answer as to which plasmid type imposes a higher fitness cost, the mechanism of cost mitigation by *hns* is noteworthy because it does not rely on host-specific factors and thus could be effective across a wide range of hosts. This perspective aligns with our hypothesis that the *hns*-centered strategy is compatible with both NHR and BHR, whereas the psiB-centered strategy is effective only for NHR plasmids, a model strongly supported by their contrasting distributions: *hns* was found in 30% of NHR and 60% of BHR plasmids, whereas *psiB* was present in 40% of NHR plasmids but was completely absent from BHR plasmids.

Importantly, both H-NS and PsiB plasmids harbored significantly more ARGs than plasmids lacking either gene, suggesting that both the stealth and manipulative strategies promote ARG dissemination (Fig. 3a). However, they do so in distinct ways: H-NS plasmids, with their broad host range, appear to act as early hubs that acquire novel ARGs from diverse environments, whereas PsiB plasmids contribute to the amplification of already prevalent ARGs within narrower ecological niches. This “stealth-first” model recapitulates historical ARG dissemination patterns (Fig. 4a). We further observed that integrons may contribute to the acquisition of ARGs by H-NS plasmids. Although *hns* is known to enhance integrase expression while repressing the transcription of cassette genes [62], it has remained unclear whether its overall effect on integron function is beneficial or detrimental. Our results suggest that, at least in the context of ARG acquisition, the regulatory characteristics of *hns*—or other features associated with H-NS plasmids—are compatible with, and may even promote integron-mediated gene capture. These results are consistent with recent studies linking features of plasmids to the dissemination of ARGs—for example, the idea that insertion sequences carried on plasmids drive the evolution of novel resistances (Sastre-Dominguez, J. *et al.*, bioRxiv, doi:10.1101/2025.08.12.669853) and that highly mobile, BHR plasmids with rapid turnover of accessory gene repertoires are especially prone to spread ARGs [63]. At the same time, our analysis extends this literature by introducing a complementary perspective: the role of distinct plasmid survival strategies.

These results clarify how conjugative plasmids and their survival strategies partition into two types linked to host-range regimes and contribute to ARG dissemination in distinct ways. However, our approach has limitations. First, while our study establishes *hns* as a marker for a major class of BHR plasmids, this focus may underrepresent other BHR lineages that achieve a broad host range through alternative mechanisms. For example, although DUF3085 is encoded by only a small fraction of BHR plasmids, it is the most strongly BHR-enriched factor and shows a co-occurrence pattern distinct from *hns* (Supplementary Fig. S4a), so analyses centered on H-NS plasmids could miss the potential strategy enabled by this protein. Although the function of DUF3085 is unknown, we found that it is frequently encoded adjacent to RadC—a putative nuclease implicated in DNA competence [64, 65]—and AlphaFold3 multimer predictions support the possibility that these proteins form a complex (Supplementary Fig. S6). Second, in this study, gene annotation was performed by assigning to each CDS the single Pfam domain with the lowest *E*-value in hmmscan. While this best-hit approach simplifies downstream analyses, it can obscure biologically meaningful complexity: (i) genes that share a Pfam label but comprise functionally diverse subfamilies, and (ii) multi-domain (“polyvalent”) proteins that harbor several independent modules within a single polypeptide. The latter may include proteins that aggregate anti-defense domains, potentially contributing to plasmid adaptation [66]. We therefore regard our annotations as conservative and note as a limitation that our pipeline may undercount multi-domain architectures and mask functional heterogeneity. Third, our analysis focused on plasmids from bacterial isolates. To assess the impact of this bias, we examined 11 160 IMG/PR metagenome-assembled, putative conjugative plasmids (Supplementary Fig. S8). In this dataset, H-NS plasmids (*n* = 367) and PsiB plasmids (*n* = 74) were infrequent, and plasmids encoding both genes were rare (*n* = 3). Consequently, while a trend toward mutual exclusion was robustly observed, it did not reach statistical significance owing to the predominance of “None” plasmids (*n* = 10 716), which mainly originate from taxa outside of Enterobacterales (only 393 plasmids out of 10 716 “None” plasmids originate from Enterobacterales). This result implies that our marker-based approach may not capture survival strategies of plasmids originating outside Enterobacterales, consistent with the taxonomic distribution obtained from InterPro web server [67] showing that *hns* (PF00816.24) and *psiB* (PF06290.14) are predominantly restricted to Pseudomonadota. Whether similar evolutionary differentiation into stealth versus manipulative strategies exists in other lineages—and which genes might drive such divergence—remains an intriguing question for future research.

Evolutionary predictability has been demonstrated through the recurrent emergence of beneficial mutations across lineages [68], and forecasting the evolution of antibiotic resistance remains an urgent challenge [69]. MGEs, which mediate horizontal gene transfer, are central to the spread of resistance genes [70]. While ARG acquisition is beneficial under antibiotic pressure, it imposes a cost in its absence—making its dynamics context-dependent and difficult to model. Recent approaches incorporate gene transfer networks and genome-wide acquisition/loss patterns to better understand HGT-prone ARGs [70, 71]. Complementing this line of research, our study provides insights from the MGE perspective by identifying characteristic features of plasmids that are prone to acquiring and disseminating ARGs. Here, we identify a generalizable signature of ARG dissemination: ARGs consistently emerge first on “stealth” MGEs before appearing on others. This temporal pattern offers a generalizable framework for inferring the stages of ARG dissemination, with stealth-exclusive ARGs representing early-stage variants and those found across multiple MGE types reflecting broader epidemiological spread. Together, these findings lay a foundation for predictive frameworks that integrate MGE biology into the surveillance and control of antimicrobial resistance.

## Supplementary Material

gkaf1479_Supplemental_Files

## Data Availability

The data underlying this article are available within the article and its online supplementary material. Additional supporting data are available in the Figshare repository at 10.6084/m9.figshare.28923875.
