# Supplementary material for: Host range and antibiotic resistance dissemination are shaped by distinct survival strategies of conjugative plasmids
Source: Nucleic Acids Res. 2026 Jan 23;54(2):gkaf1479. doi: 10.1093/nar/gkaf1479 (PMC12826799; doi:10.1093/nar/gkaf1479)
Supplement: gkaf1479_Supplemental_Files [file gkaf1479_supplemental_files.zip › Ono_etal_Supplementary_materials.doc]

**Supplementary material**

**Host range and antibiotic resistance dissemination are shaped by distinct survival strategies of conjugative plasmids**

Ryuichi Ono^1,2,3,^*, Naoki Konno^4,5,6,^*, Yuki Nishimura^3^, and Chikara Furusawa^5,7^

^1^ Innovative Genomics Institute, University of California, Berkeley, Berkeley, CA 94720, USA

^2^ Department of Comparative Biochemistry, University of California, Berkeley, Berkeley, CA 94720, USA

^3^ Department of Integrated Biosciences, Graduate School of Frontier Sciences, the University of Tokyo, Kashiwa, Chiba, 277-0882, Japan

^4^ Department of Biological Sciences, Graduate School of Science, the University of Tokyo. Bunkyo-ku, Tokyo, 113-0032, Japan
^5^ Universal Biology Institute, The University of Tokyo, 7-3-1 Hongo, Bunkyo-ku, Tokyo 113-0033,Japan

^6^ Department of Genetics, School of Medicine, Stanford University, 240 Pasteur Drive, Palo Alto, CA, 94304, USA

^7^ Center for Biosystems Dynamics Research, RIKEN, 6-7-1 Minatojima-minamimachi, Chuo-ku, Kobe, 650-0047, Japan

* To whom correspondence should be addressed. Correspondence may also be addressed to Naoki Konno. Tel: +1-510-944-2242 (R.O.), +81-3-5841-4512 (N.K.); Fax: Not Available; Email: ryuichi@berkeley.edu (R.O.), konno-naoki555@g.ecc.u-tokyo.ac.jp (N.K.)

**SUPPLEMENTARY DATA**

**Supplementary Figs**

**Supplementary Fig. 1: Filtering of genes used in the enrichment analysis. a,** Distribution of Simpson’s index for genes detected in NHR plasmids. Simpson’s index was calculated based on the MOB types of NHR plasmids encoding each gene. The histogram (left) shows the distribution of Simpson’s index values, while the plot on the right illustrates the proportion of genes with Simpson’s index values exceeding a given threshold. **b,** Distribution of Simpson’s index for genes detected in BHR plasmids, along with the corresponding plot showing the proportion of genes with Simpson’s index values exceeding a given threshold. The mean value is indicated by a red dotted line.

**Supplementary Fig. 2**: **Distribution of Jaccard Index.** Histogram showing the distribution of Jaccard index values for the 990 gene pairs in which both genes are encoded by ≥250 plasmids. The Jaccard index was calculated based on the sets of plasmids encoding each gene. The x-axis represents the Jaccard index, while the y-axis indicates the number of gene pairs in each bin. The bin contains the *h-ns* and *psiB* pair (Jaccard index = 4.78 × 10⁻³), which ranked as the 17th smallest, is highlighted in red.

**Supplementary Fig. 3: The anti-co-occurrence of h-ns and psiB is robust to phylogenetic correction. a,** The dataset was subsampled to one representative plasmid per Plasmid Taxonomic Unit (PTU). The contingency table shows the counts of plasmids with *h-ns* only, *psiB* only, both, or neither gene. The mutually exclusive pattern remains statistically significant (Chi-squared test, *p* = 0.035). **b,** The anti-co-occurrence pattern also persists within individual major MOB groups. Pie charts show the proportion of H-NS, PsiB, Both, and None plasmids for the five most abundant MOB types among the 943 plasmids used for the initial enrichment analysis. Significance is shown with asterisks (**p* < 0.05; *****p* < 0.0001, Chi-squared test).

**Supplementary Fig. 4: Pairwise co-occurrence structure among genes in BHR or NHR plasmids. a, b,** Heatmap showing pairwise dissimilarity (1 − Jaccard index) among the top 10 genes enriched in BHR and NHR plasmids, respectively. Hierarchical clustering was performed using average linkage.

**Supplementary Fig. 5**: **Gene-neighborhood analysis around DUF3085.** Histogram showing the mean distance (in CDS steps) from DUF3085. For each of the 82 plasmids that encode DUF3085 in our dataset, we computed for every CDS on the same plasmid the minimal gene-order distance to DUF3085 (strand ignored; wrap-around allowed; see Methods). CDSs were then grouped by their gene annotation, and the mean distance to DUF3085 was calculated for each gene. The x-axis shows these per-gene means; the y-axis shows the number of genes per bin. The red dotted line marks the mean of the distribution.

**Supplementary Fig. 6: Co-folding of DUF3085 with neighboring proteins.** For genes whose mean gene-order distance from DUF3085 was ≤10 CDSs in our dataset (n = 9; selected in Supplementary Fig. 5), full-length protein pairs—DUF3085 and the co-encoded partner on the same contig—were submitted to the AlphaFold3 web server (33) for heteromeric two-chain prediction using default settings; the top-ranked model for each pair is shown. The accession numbers for the protein sequences used in this analysis are listed in Supplementary Table 4.

**Supplementary Fig. 7: ARG prevalence categories and diversity across plasmid types. a, b,** Characteristics of ARGs belonging to each prevalence category; ‘Rare’ (<10 plasmids), ‘Moderate’ (10–99), and ‘Prevalent’ (≥100). For each ARG, Simpson’s index over MOB types was calculated and compared between prevalence categories. Significance is shown with asterisks (****FDR < 0.0001, Mann–Whitney U test). **a,** Analysis on all detected ARGs. **b,** Analysis on ARGs located within integron regions.

**Supplementary Fig. 8: Filtering and preliminary characterization of metagenome-derived conjugative plasmids (IMG/PR). a,** Contingency tables showing the distribution of *h-ns* and *psiB* across metagenome-derived conjugative plasmids in IMG/PR dataset. The numbers within the boxes indicate the count of plasmids in each category, with color intensity corresponding to plasmid counts. Significance is shown with asterisks (****p < 0.0001, Chi-squared test). **b,** Characteristics of each plasmid type. Dot colors correspond to plasmid MOB types, with color assignments matching those in Fig. 2b. Significance is shown with asterisks (****FDR < 0.0001, Mann–Whitney U test).

**Supplementary Fig. 9: Complete plasmid–protein-cluster network based on proteome similarity.** Bipartite network linking plasmids to homologous protein clusters (HPCs) encoded on them. Plasmids are shown as colored nodes and HPCs as white nodes; an edge connects a plasmid to an HPC when the plasmid encodes ≥1 protein assigned to that cluster. The layout was computed with ForceAtlas2 (see Methods), which places plasmids that share many HPCs closer together. **a,** Plasmids colored by plasmid type (H-NS, PsiB, Both, None). **b,** Same network with plasmids colored by MOB family. The area circled by the red rectangle corresponds to the area shown in Fig. 2b.

**Supplementary Fig. 10: ARG acquisition by H-NS plasmids has preceded that by PsiB plasmids throughout the historical spread of prevalent ARGs.** Cumulative frequency curves showing the temporal increase in plasmid records encoding the top 10 most prevalent ARGs, based on collection dates from the PLSDB database.

**Supplementary Discussion 1: Extended explanation and example of quantifying gene distribution diversity across MOB types using Simpson’s index**

Let be the total number of BHR (or NHR) plasmids carrying gene. If is the number of such plasmids in MOB group, define.
Simpson’s index is, where.

As a more detailed example, suppose that gene is encoded in 30 BHR MOBP plasmids and 5 BHR MOBF plasmids. In this case, Simpson’s index will be calculated as follows;

In the enrichment analysis conducted in this research, since we used a threshold of, gene in this example will be excluded from the downstream analysis. This threshold was determined empirically by examining the distribution of Simpson's index values for all genes in our dataset (Supplementary Fig. 1). We observed a clear drop-off in the number of genes with an index value above 0.4, suggesting this value serves as a natural cutoff to distinguish between genes confined to a narrow set of MOB types and those that are more broadly distributed across diverse plasmid lineages.

**Supplementary Discussion 2: Roles of *h-ns* beyond transcriptional regulation of hosts chromosome**

Beyond regulating chromosomal transcription, *h-ns* can act as a xenogeneic silencer that preferentially represses AT-rich foreign DNA (1, 2), and has been implicated in modulating transposon insertion frequency (3). Importantly, plasmid-encoded *h-ns* exhibits properties that are not simply substitutable by chromosomal *h-ns*: regulation of conjugation by plasmid-borne *h-ns* cannot be complemented by the chromosomal paralogue (4), and plasmid-borne *h-ns* proteins form a deep-branching clade distinct from chromosomal *h-ns* (4). Moreover, In recipients, plasmid-encoded *h-ns* reduces the fitness cost of acquisition: by pre-binding AT-rich sites on the plasmid, it limits titration of chromosomal *h-ns* to the incoming plasmid and thereby blunts genome-wide transcriptional shifts; accordingly, plasmids with *h-ns* confer higher recipient fitness than counterparts without *h-ns* (5). In addition, transcriptional silencing of the plasmid by plasmid-encoded *h-ns* has been proposed to minimize acquisition-associated toxicity by preventing sequestration of RNA polymerase on the plasmid (6, 7).

**Supplementary References**

1. Navarre,W.W., Porwollik,S., Wang,Y., McClelland,M., Rosen,H., Libby,S.J. and Fang,F.C. (2006) Selective silencing of foreign DNA with low GC content by the H-NS protein in Salmonella. *Science*, 313, 236–238.

2. Navarre,W.W., McClelland,M., Libby,S.J. and Fang,F.C. (2007) Silencing of xenogeneic DNA by H-NS-facilitation of lateral gene transfer in bacteria by a defense system that recognizes foreign DNA. *Genes Dev.*, 21, 1456–1471.

3. Cooper,C., Legood,S., Wheat,R.L., Forrest,D., Sharma,P., Haycocks,J.R.J. and Grainger,D.C. (2024) H-NS is a bacterial transposon capture protein. *Nat. Commun.*, 15, 7137.

4. Wang,A., Cordova,M. and Navarre,W.W. (2025) Evolutionary and functional divergence of Sfx, a plasmid-encoded H-NS homolog, underlies the regulation of IncX plasmid conjugation. *MBio*, 16, e0208924.

5. Doyle,M., Fookes,M., Ivens,A., Mangan,M.W., Wain,J. and Dorman,C.J. (2007) An H-NS-like stealth protein aids horizontal DNA transmission in bacteria. *Science*, 315, 251–252.

6. San Millan,A. and MacLean,R.C. (2017) Fitness costs of plasmids: A limit to Plasmid transmission. *Microbiol. Spectr.*, 5.

7. Lamberte,L.E., Baniulyte,G., Singh,S.S., Stringer,A.M., Bonocora,R.P., Stracy,M., Kapanidis,A.N., Wade,J.T. and Grainger,D.C. (2017) Horizontally acquired AT-rich genes in Escherichia coli cause toxicity by sequestering RNA polymerase. *Nat. Microbiol.*, 2, 16249.
